# An investigation of BMP-7 mediated alterations to BMP signalling components in human tenocyte-like cells

**DOI:** 10.1038/srep29703

**Published:** 2016-07-11

**Authors:** Franka Klatte-Schulz, Gerry Giese, Christopher Differ, Susann Minkwitz, Karen Ruschke, Regina Puts, Petra Knaus, Britt Wildemann

**Affiliations:** 1 Julius Wolff Institute, Charité-Universitaetsmedizin Berlin, Germany; 2 Berlin-Brandenburg Center for Regenerative Therapies, Charité-Universitaetsmedizin Berlin, Germany; 3 Berlin-Brandenburg School for Regenerative Therapies, Charité-Universitaetsmedizin Berlin, Germany; 4 Institute for Chemistry and Biochemistry, Freie Universitaet Berlin, Germany

## Abstract

The incidence of tendon re-tears post-surgery is an ever present complication. It is suggested that the application of biological factors, such as bone morphogenetic protein 7 (BMP-7), can reduce complication rates by promoting tenogenic characteristics in *in vitro* studies. However, there remains a dearth of information in regards to the mechanisms of BMP-7 signalling in tenocytes. Using primary human tenocyte-like cells (hTLCs) from the supraspinatus tendon the BMP-7 signalling pathway was investigated: induction of the BMP associated Smad pathway and non-Smad pathways (AKT, p38, ERK1/2 and JNK); alterations in gene expression of BMP-7 associated receptors, Smad pathway components, Smad target gene (ID1) and tenogenic marker scleraxis. BMP-7 increases the expression of specific BMP associated receptors, BMPR-Ib and BMPR-II, and Smad8. Additionally, BMP-7 activates significantly Smad1/5/8 and slightly p38 pathways as indicated by an increase in phosphorylation and proven by inhibition experiments, where p-ERK1/2 and p-JNK pathways remain mainly unresponsive. Furthermore, BMP-7 increases the expression of the Smad target gene ID1, and the tendon specific transcription factor scleraxis. The study shows that tenocyte-like cells undergo primarily Smad8 and p38 signalling after BMP-7 stimulation. The up-regulation of tendon related marker genes and matrix proteins such as Smad8/9, scleraxis and collagen I might lead to positive effects of BMP-7 treatment for rotator cuff repair, without significant induction of osteogenic and chondrogenic markers.

High rates of re-tears or non-healing represent the common complications after surgical intervention to repair the rotator cuff tendons. Modified suture techniques, aimed at improving tendon strength after surgical repair have not resulted in distinctly better tendon healing outcomes for the patients^1^,^2^. There is a point of view that the investigation and optimisation of biological treatment strategies will reduce future rates of re-tears and non-healing post-surgery. One such biological strategy would be the application of growth factors, such as bone morphogenetic protein-7 (BMP-7), to improve the healing outcome after tendon surgery. The *in vitro* application of BMP-7 to tenocytes improved cell activity, and the expression and synthesis of extracellular matrix proteins such as collagen-I and III, without enhanced expression of collagen-II and osteocalcin^3^.

BMP-7 functions through the binding to specific BMP transmembrane receptors at the cell surface. These specific BMP-receptors (BMPRs) are classified into two groups, BMPR type I and BMPR type II, and conduct BMP signalling through the formation of a heterotetrameric complex comprised of two receptors of BMPR type I and BMPR type II each^10^,^11^. The BMPR type I receptors: BMPR-1a, BMPR-1b and ActR-1; and the BMPR type II receptors: BMPR-II: ActR-IIa and ActR-IIb have been shown to be associated with BMP-7 signalling^12^.

Activated receptors induce intracellular signalling through Smad and/or Smad-independent pathways (phosphoinositide-3-kinase (PI3K)/ protein kinase B (Akt) pathway, the Jun nuclear kinase (JNK)/p38 pathway and the extracellular signal regulated kinase (ERK))^13^.

The Smad pathway is initiated by binding of a BMP molecule to the receptor complex resulting in a phosphorylation of the receptor regulated Smad1/5/8 (rSmads). The phosphorylated rSmads are released to the cytoplasm as a dimer and/or trimer to form a complex with the mediator Smad4 (co-Smad4). This Smad complex translocates into the nucleus, where it activates the transcription of target genes, e.g. inhibitor of differentiation 1 (ID1)^14^,^15^. On the other hand the Smad-independent pathways are activated by phosphorylated BMP bound receptor complex^12^ and act via Akt^16^,^17^, Erk1/2^18^,^19^, p38^20^,^21^, JNK^18^,^22^ and Rho/ROCK^23^ pathways.

BMP signalling mechanisms are well studied and vary depending on the BMP ligand and on the cell type. To date, no detailed BMP-7 signalling analysis regarding receptors, signalling molecules and target genes was performed in human tenocytes, which is to be addressed in this present study. An improved understanding of BMP signalling mechanisms in tenocytes may open opportunities to use this growth factor for future treatment strategies to improve tendon healing.

## Results

### Basal gene expression level of tenocyte-like cells

Primary human tenocyte-like cells (hTLCs) were isolated from the supraspinatus tendon from six male donors (62–67 years, Goutallier grade 1 or 2). Gene expression was evaluated by quantitative real time polymerase chain reaction (qRT-PCR). All hTLCs expressed tenocyte markers or typical proteins such as scleraxis, mohawk, collagen I, and aggrecan, whereas the osteogenic makers Runx2 and osterix were not expressed and osteocalcin only to a lesser extent. Collagen II as the main ECM component of cartilage was not expressed and Sox9 only in very small amounts (Table 1).

### Basal gene expression level of BMP receptors and Smads in untreated hTLCs

The basal gene expression level of the type I receptors showed the strongest presence of BMP receptor (BMPR)-Ia, closely followed by Activin receptor (ActR)-I in the tenocyte-like cells (TLCs). BMPR-Ib was expressed in lower amounts. In the type II receptor group, BMPR-II was expressed in high amounts, while ActR-IIa and ActR–IIb showed weaker expression. The receptor regulated Smad5 was expressed in high amounts followed by Smad8 and 1 (Table 1).

### BMP receptor expression in hTLCs after BMP-7 stimulation

**Figure 1:**
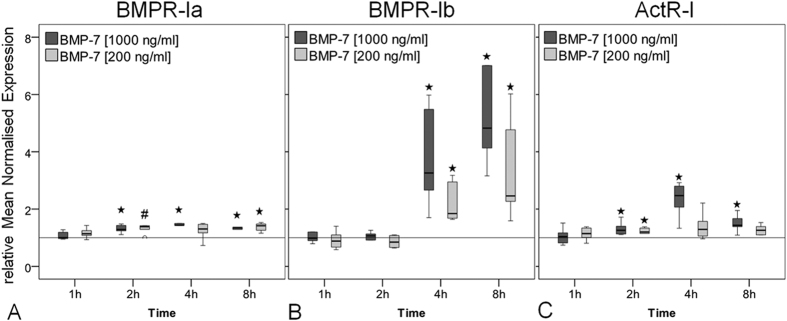
Gene expression of BMP type I receptors in response to BMP-7. Box plots represent qRT-PCR results given as mean normalised expression relative to 18S rRNA and the untreated control (reference line) using an efficiency corrected formula (n = 6). Symbols indicate statistically significant differences to the untreated control. (**A)** BMPR-Ia expression was increased after 2 to 8 h of BMP-7 stimulation (^#^p = 0.015, *p = 0.002). (**B)** BMPR-Ib expression was highly increased after 4 and 8 h of stimulation compared to unstimulated control (*p = 0.002). (**C)** ActR-I expression was increased at 2 h to 8 h with a peak at 4 h in the high BMP-7 group (*p = 0.002).

The hTLCs were treated for 1, 2, 4 and 8 hours with two concentrations of BMP-7 (200 ng/ml and 1000 ng/ml). Of the type I receptor group, BMPR-Ia expression was significantly increased from 2 hours after low (200 ng/ml) and high (1000 ng/ml) BMP-7 stimulation compared to untreated control (200 ng/ml BMP-7 at 2 h: p = 0.015, others: p = 0.002, Fig. 1A). Expression of BMPR-Ib showed the strongest regulation, which was significantly up-regulated at 8 hours stimulation with a 4.8-fold at the high and 2.5-fold at the low BMP-7 concentration compared to control (all p = 0.002, Fig. 1B). The ActR-I expression increased significantly after 2 hours of stimulation at the high concentration of BMP-7 (all p = 0.002, Fig. 1C).

**Figure 2:**
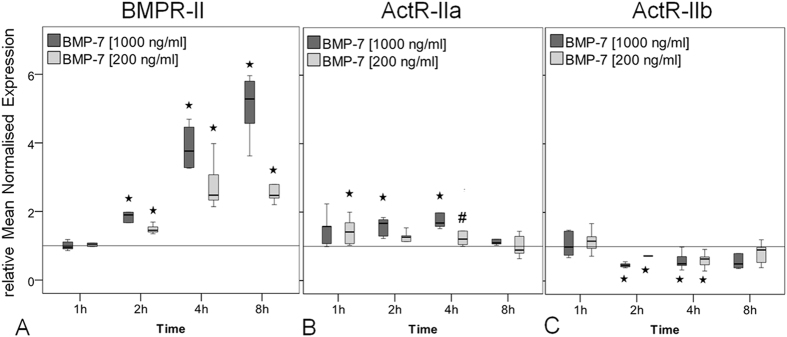
Gene expression of BMP type II receptors in response to BMP-7. Box plots represent qRT-PCR results given as mean normalised expression relative to 18S rRNA and the untreated control (reference line) using an efficiency corrected formula. Symbols indicate significant differences to the untreated control (n = 6). (**A)** BMPR-II expression was strongly increased after 2 to 8 h of BMP-7 stimulation which was more pronounced in the high BMP-7 group (*p = 0.002. (**B)** The expression of ActR-IIa was increased in the low BMP-7 group after 1 h, in the high BMP-7 group after 2 h and 4 h after stimulation by both concentrations (^#^p = 0.03, *p = 0.004). (**C)** ActR-IIb expression was significantly down regulated after 2 h and 4 h compared to the untreated control (*p = 0.002).

Of the type II BMP receptors: BMP-7 stimulation induced the strongest increase to BMPR-II gene expression. Expression increased significantly from 2 hours, to reach a maximum increase at 8 hours of 5.3-fold at high concentration, and 2.5-fold for the low concentration (all p = 0.002, Fig. 2A). ActR-IIa expression was slightly but significantly increased at 1, 2, 4 hours of stimulation (200 ng/ml BMP-7: p = 0.03, others: p = 0.004, Fig. 2B) but no increase was measured at 8 hours. The ActR-IIb gene expression significantly decreased 2 and 4 hours after stimulation in both concentrations of BMP-7 compared to the unstimulated control (all p = 0.002, Fig. 2C).

### Phosphorylation of Smad1/5/8 in hTLCs stimulated with BMP-7

**Figure 3:**
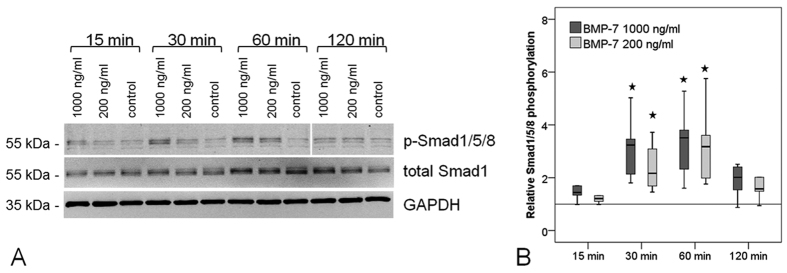
Phosporylation of Smad1/5/8 after BMP-7 stimulation. (**A)** Exemplary western blots show increased Smad 1/5/8 phosphorylation after 30 and 60 min of stimulation with BMP-7, but no increase in total Smad1 levels compared to unstimulated controls. GAPDH serves as reference protein. Imaging was conducted using the Odyssey imager and LiCor Odyssey software. (**B)** Quantification of relative phosphorylation normalised to total Smad1 and GAPDH was significantly increased at 30 and 60 min (n = 6). Stars mark significant differences to the untreated control, p = 0.002.

The C-terminal phosphorylation of Smad1/5/8 in hTLCs after 15, 30, 60 or 120 minutes of BMP-7 stimulation was determined by the use of phosphorylation state-specific antibodies. Quantification was achieved when normalising the signal to total Smad1 and GAPDH. BMP-7 induced significant phosphorylation of Smad 1/5/8 after 30 and 60 min of stimulation (all p = 0.002) and the signal declined again at 120 min (Fig. 3).

### Regulation of Smad expression in response to BMP-7 stimulation in hTLCs

**Figure 4:**
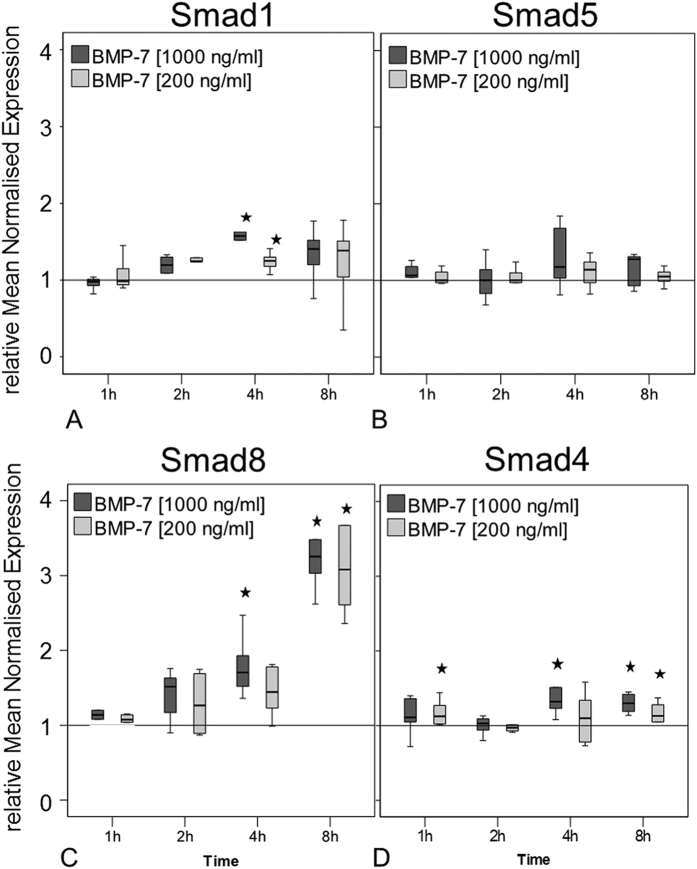
Gene expression of Smad1, 5 and 8/9 and co-Smad4. Box plots represent qRT-PCR values given as mean normalised expression relative to 18S rRNA and the untreated control (reference line) using an efficiency corrected formula (n = 6). Stars indicate significant differences to the untreated control. (**A)** Expression of Smad1 was increased only at 4 h of BMP-7 stimulation (*p = 0.002). (**B)** Smad5 expression was not regulated compared to unstimulated control. (**C)** Smad8/9 expression increased after 4 h in the high BMP-7 group and highly increased after 8 h in both concentrations (*p = 0.002). (**D)** Smad4 expression was slightly increased after 1, 4 and 8 h of stimulation (*p = 0.002).

To determine if BMP-7 is inducing alterations in expression of main signalling components the gene expression of receptor regulated Smad1, 5, 8 and the co-Smad4 in the hTLCs was investigated. The expression of the receptor regulated Smad1 was significantly elevated at 4 hours after stimulation by both BMP-7 concentrations (all p = 0.002), while Smad5 expression was not altered (Fig. 4A,B). The expression of the receptor regulated Smad8 (whose gene is called Smad9) increased significantly at later stimulation times (4 and 8 hours) with a maximum increase of around 3-fold compared to the untreated control (all p = 0.002, Fig. 4C). The co-Smad4 expression was slightly increased over most stimulation times (all p = 0.002, Fig. 4D).

### Non-Smad signalling induction by BMP-7 in hTLCs

**Figure 5:**
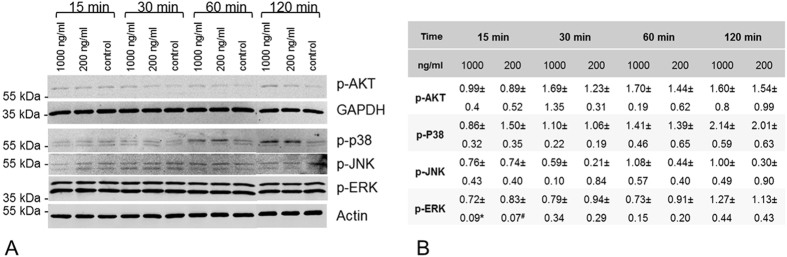
Phosphorylation of non-Smad signalling molecules. (**A)** Exemplary western blots show increased p38 phosphorylation after 60 and 120 min. p-AKT was slightly increased after 120 min. No regulations were visible for p-Erk1/2 and p-JNK. Beta actin and GAPDH served as reference proteins. Imaging was conducted using the Odyssey imager and LiCor Odyssey software. (**B)** Quantification of 3 independent western blots (mean ± SD). Bands from the protein of interest were normalised to β-Actin or GAPDH and the unstimulated control, *p = 0.003, ^#^p = 0.031, compared to control.

Phosphorylated non-Smad signalling proteins, i.e. Erk1/2, p38, JNK and AKT were evaluated in 3 exemplary hTLCs after BMP-7 stimulation. A slight increase in the level of phosphorylated p38 was seen after 15 minutes of BMP-7 (200 nM) stimulation and was increased after 60 and 120 minutes with both concentrations. BMP-7 induced an increase in phosphorylated AKT after 30–120 minutes. Phosphorylated ERK1/2 was abundant in high concentration independent of the stimulation and p-JNK proteins were not upregulated (Fig. 5).

### Target gene expression in hTLC

**Figure 6:**
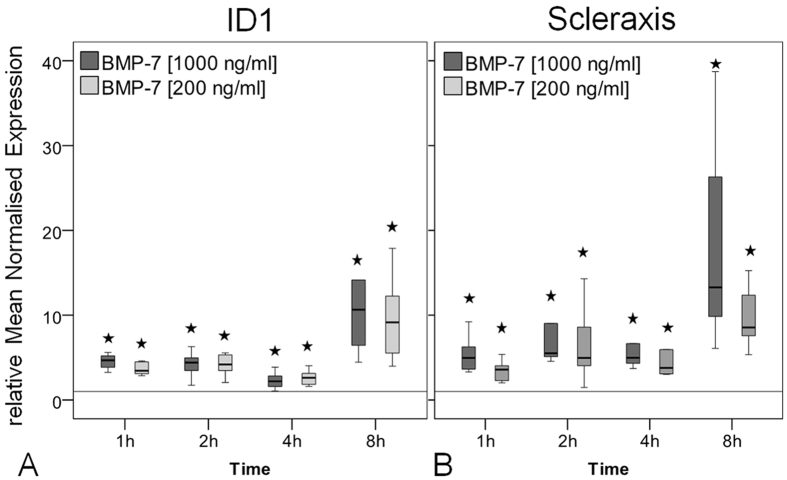
Gene expression of BMP target gene ID1 and tendon marker scleraxis. Box plot graphs represent qRT-PCR values given as mean normalised expression relative to 18S rRNA and the untreated control (reference line) using an efficiency corrected formula (n = 6). Stars indicate significant differences to the untreated control. (**A)** ID1-expression was significantly increased at all concentrations and time points with the strongest increase at 8 h compared to the unstimulated control (*p = 0.002). (**B)** The expression of scleraxis was up-regulated at both concentrations for all time points and showed the strongest increase after 8 h of BMP-7 stimulation (*p = 0.002).

The Inhibitor of Differentiation 1 (ID1) is a primary target gene in the Smad-signalling pathway. Stimulation of hTLC with BMP-7 significantly increased the ID1 expression compared to the untreated control with a maximum increase of 10.7-fold in the high and 9.2-fold in the low BMP-7 dose group at 8 hours of stimulation (all p = 0.002, Fig. 6A). The expression of the tendon related transcription factor scleraxis significantly increased over the entire stimulation period reaching a maximum increase of 13.3-fold for the high and 8.5-fold for the low concentration group at the late time point (all p = 0.002, Fig. 6B).

**Figure 7:**
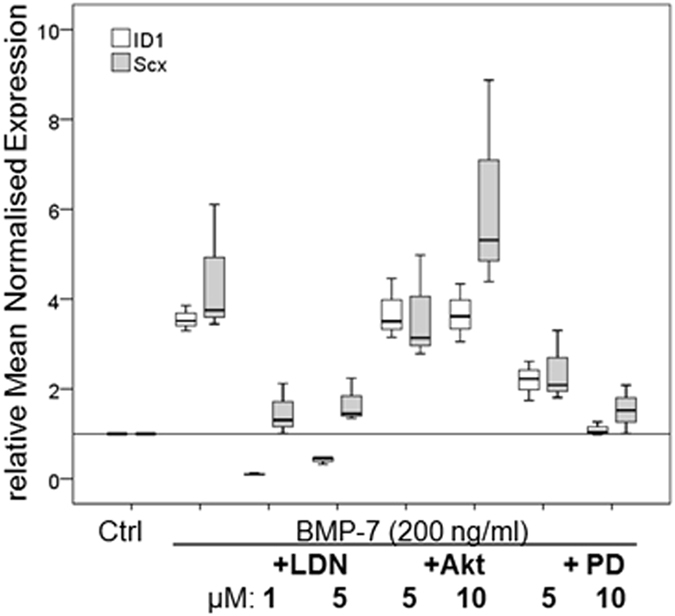
Inhibition of intracellular pathways, gene expression of BMP target gene ID1 and tendon marker scleraxis. Box plot graphs represent qRT-PCR values given as mean normalised expression relative to 18S rRNA and the untreated control (reference line) using an efficiency corrected formula (n = 3). Inhibitors for the specific pathway were used in two concentrations. The inhibition of the Smad-pathway by LDN-193189 (LDN) resulted in a significant reduction of ID1 and scleraxis expression, less reduction was seen after inhibition of the p38 pathway with PD169316 (PD) and no effect after inhibition of the Akt-pathway (Akt), *p < 0.003, ^#^p < 0.04, compared to BMP-7.

Pathway inhibition experiments showed that the enhancing effect of BMP-7 on the expression of ID-1 and scleraxis can be reversed by LDN-193189 (inhibitor of Smad-pathway) and PD169316 (inhibitor of p38-pathway) but not by the AKT-inhibitor (Fig. 7).

**Figure 8:**
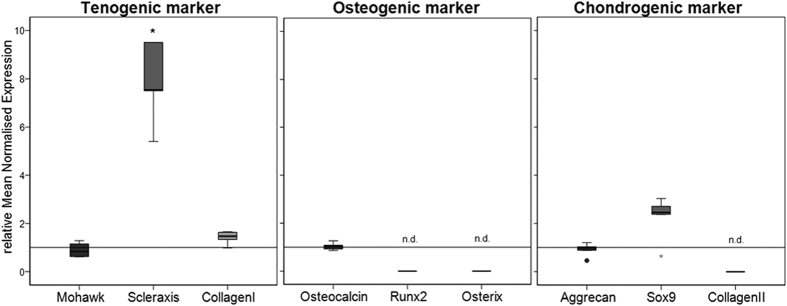
Gene expression of tenogenic, osteogenic, and chondrogenic markers to evaluate cell phenotype after BMP-7 stimulation. Box plot graphs represent qRT-PCR values given as mean normalised expression relative to 18S rRNA and the untreated control (reference line) using an efficiency corrected formula (n = 6). Stars indicate significant differences to the untreated control. After 8 h of BMP-7-stimulation hTLCs expressed significantly higher amounts of the tenogenic markers scleraxis (p = 0.008) and matrix protein collagen I, whereas osteogenic markers were not upregulated or not expressed at all (Runx2 and osterix) and only Sox9 was upregulated as a marker for chondrogenic differentiation.

To examine whether the BMP-7 stimulation of the hTLCs might alter the tenogenic to an osteogenic or chondrogenic phenotype, the expression of the tenocyte markers scleraxis, mohawk and the main matrix protein collagen I was again tested after 8 h of stimulation as well as osteocalcin, osterix, Runx2 (osteogenic marker) and Sox9, collagen type II, and aggrecan (chondrogenic marker or matrix proteins). The expression level of mohawk, which was highly expressed already in the untreated control, did not change after the BMP-7 stimulation, whereas collagen I (1.5-fold) and scleraxis (7.5-fold, p = 0.008) level increased. Runx2 and osterix expression could not be observed in the tenocyte cultures and osteocalcin expression was weak. Furthermore, collagen II expression was not detectable, the expression level of aggrecan did not change, and only Sox9 was slightly upregulated after BMP-7 stimulation (Fig. 8).

## Discussion

As unsatisfactory healing outcomes post tendon surgery remains a severe clinical complication, it is necessary to investigate the application of growth factors as treatment option for improved tendon regeneration. The growth factor BMP-7 can be considered a candidate factor as previous studies have shown that it is capable of inducing proliferation and collagen-I synthesis of cells from tendons and ligaments^3^. It is the aim of this study to further investigate BMP-7 signalling in tenocytes through the analysis of the signalling components in human tenocyte like cells (hTLCs) in the presence of BMP-7.

The present study shows for the first time that BMP-7 leads to an increase in phosphorylation of rSmads in hTLCs. The phosphorylated rSmad should complex with the co-Smad4, translocate into the nucleus and increase the expression of the transcription of ID1 (a canonical target gene in BMP signalling) and the tendon related transcription factor scleraxis. An increase of the gene expression of the rSmad component, Smad8, but no change amongst its Smad1/5 and co-Smad-4 partners is observed. Furthermore, the receptors BMPR-Ib and BMPR-II gene expression is strongly increased in hTLCs in the presence of BMP-7. Taken together this could be indicative of BMP-7 selecting a pool of signalling components (receptors and intracellular components) that are pro-tenogenic.

Different BMP ligands were shown to have different affinities for different BMP receptors. While BMP-2 preferentially binds to BMPR-Ia and –Ib and less to BMPR-II^24^,^25^, BMP-7 has high affinity for ActR-I, BMPR-Ib, and to a lesser extent BMPR-Ia^10^,^11^. BMPs have a general lower affinity for type II receptors. However, it was shown that BMP-7 also binds to BMPR-II, ActR-IIa, and ActR-IIb^26^,^27^,^28^,^29^. In COS-1 cells stimulated with BMP-7 BMPR-II has been shown to preferentially complex with ActR-I and to a lesser extent with BMPR-Ia and-Ib^28^. As the present study investigated mRNA expression levels of BMP receptors, further research needs to be carried out to investigate the receptor complex preferences of BMP-7 in hTLCs. Based on our data, showing a strong increase in BMPR-Ib and BMPR-II expression after BMP-7 stimulation, we speculated that this may increase the presence of these receptors on the cell surface and therefore increases the likelihood of BMP-7 binding a BMPR-Ib and BMPR-II receptor complex on hTLCs.

The receptor regulated Smads1/5/8 are phosphorylated through the activation of type I receptors^30^, which could possibly be BMPR-Ib in the present study. The western blot analysis showed BMP-7 stimulation of hTLCs leads to the phosphorylation of Smad1/5/8, strongly suggesting BMP-7 induces the Smad signalling cascade. The phosphorylation analysis was not able to distinguish between the three receptor-associated Smads, but the expression analysis showed a strong increase of Smad8 expression in the hTLCs, whereas, Smad1 was only slightly increased and Smad5 was not affected. This leads us to consider that Smad8 may be a prominent signalling molecule in hTLCs in response to BMP-7 treatment. This assumed preference of Smad8 signalling in the hTLC is supported by previous studies showing that Smad8 plays an important role in tenogenic differentiation^31^,^32^. It was demonstrated that Smad8 is able to induce tenogenic differentiation, while suppressing chondrogenic and osteogenic differentiation in a murine MSC cell line (C3H10T1/2) and furthermore promoted tendon formation in a rat Achilles tendon model^31^. In contrast, Smad1 and 5 were reported to induce osteogenic differentiation in C2C12 cells^33^ and C3H10T1/2 cells^31^,^34^. While the Smad1/5/8 are highly conserved: maintaining a three domain structure (MH1. MH2 separated by a linker region), the key divergence between Smad1/5 and Smad8 is found within the linker region^35^,^36^,^37^. The linker region plays an important role in post-translational modification, such as phosphorylation^38^. A previous study has shown that BMP-2 induces Smad1 and 5 phosphorylation as well as osteogenic differentiation in human tendon cells^39^. However, Smad1 and 5 were only weakly regulated in the present study, we surmise, osteogenic differentiation might not occur after BMP-7 stimulation. This is also supported by the unchanged osteocalcin expression in this and previous studies^3^. Hoffmann *et al*. clearly showed that BMPR-Ib strongly increases the phosphorylation of Smad8 as compared with other BMPR type I receptors^31^. This would be in line with the present findings of a strongly increased BMPR-Ib expression together with a strongly up-regulated Smad8 expression. A recently published study investigated the role of Smad1, Smad5, and Smad8 in BMP signalling and concluded that Smad8 has a unique action compared to the other two Smads^40^. After activation of the BMP signalling, Smad8 expression was increased in C2C12 cell line resulting in a reduced BMP activity. This present study also demonstrates an increased Smad8 expression but not a reduction in the BMP-activity after BMP stimulation was seen in the tenocyte-like cells.

In the present study, the co-Smad4 expression was slightly increased; indicating the possibility of the formation of a Smad complex in hTLCs after BMP-7 stimulation. This complex could translocate into the nucleus to activate the transcription of target genes. One of the target genes is ID1, a helix loop helix protein^41^, which expression was significantly increased by BMP-7 in the present study. The strong increase in ID1 expression, which is significantly reduced after inhibition of the Smad-pathway, furthermore underlines the utilisation of Smad signalling in the hTLCs after BMP-7 stimulation.

Our examination of the non-Smad signalling components in hTLCs in the presence of BMP-7 has shown: an increase of phosphorylated p38; a slight increase of phosphorylated Akt; while phosphorylated ERK1/2 and JNK remained mainly unaltered, except for a significant downregulation of pERK after 15 minutes. Due to the fact that only three samples were analysed, the statistic is not very strong and we would not interpret this as a dramatic effect. From this we surmise BMP-7 preferentially selects the p38 signalling pathway compared to the other non-Smad pathways investigated in this study. This is supported by the reduced ID1 expression after inhibition of the p38 kinase. These results are in accordance with a study from Fiori *et al*. demonstrating that the inhibition of p38 results in a reduced ID1 expression after BMP-4 stimulation, whereas inhibition of ERK or JNK had no effect^42^.

Despite previous studies having associated BMP-7 with the PI3K/Akt pathway^16^,^17^, it is inconclusive from our data if BMP-7 leads to Akt phosphorylation. Different studies found PI3K/Akt to have paradoxical roles in both: promoting^43^ and inhibiting^44^,^45^ osteoblastic differentiation. The BMP-7 induced inhibition of osteoblastic differentiation is mediated through induction of PI3K/Akt and could aid cell decision making towards a tenogenic outcome; essential to the promotion of tendon healing.

It is still a matter of discussion whether BMP-7 positively or negatively affects tendon healing. On one hand, BMP-7 has been described to have a negative effect on tendon healing by inducing bone formation^46^. The hTLCs in this study, however, showed no increase in osteogenic factors after stimulation with BMP-7. Only Sox9, a high-mobility group box-containing transcription factor functioning as a key regulator of chondrogenesis, was upregulated after 8 h stimulation. In a cell culture study, overexpression of Sox9 in tenocytes led to a switch of the cell profile towards the chondrogenic lineage, accompanied by a downregulation of scleraxis and an upregulation of type II collagen and Chondromodulin-I^47^. In contrast, in the present study scleraxis was significantly upregulated and collagen II was not detectable at all indicating no chondrogenic differentiation after stimulation with BMP-7. On the other hand, positive effects of BMP-7 were already reported in the promotion of tendon repair^48^. Further evidence that BMP-7 has positive effects on tendon repair has been postulated in several cell culture studies, which revealed BMP-7 enhanced cell proliferation and Col-I expression of ligament and tendon cell cultures^3^. Lui *et al*. described BMP/Smad signalling to be related to tendinopathy as investigated in a rat tendon injury model, however, BMP-7 and BMPR-II seem to have lesser involvement^49^. Furthermore, BMP-7 is shown to be intensely expressed in the developing flexor tendon^50^ and is found in ovine tendon-bone healing^51^. These positive findings are further underlined by this present study showing a strongly increased scleraxis expression in hTLCs after BMP-7 stimulation. Scleraxis is a basic-helix-loop-helix transcription factor that plays an important role in tendon development and differentiation^52^,^53^. For example, scleraxis can regulate the expression of the glycoprotein tenomodulin, which acts as another important tendon marker^54^,^55^. Tenomodulin is key to collagen fibril maturation and its loss *in vivo* leads to loss of tendon density, this may indicate the essential role of scleraxis for a proper tendon healing outcome and supports the assumption that BMP-7 might be a good treatment option for tendon repair. This assumption is underlined by previous studies of the group showing an increased cell proliferation and collagen expression and synthesis of tenocytes (mostly independent on donor characteristics such as age, sex, degeneration) after stimulation with BMP-7 for several days^3^,^4^,^5^.

## Conclusion

This present study shows that BMP-7 signalling of hTLCs, isolated from human supraspinatus tendons (donors: male, 62–67 years, Goultallier grade 1–2), operates primarily through the Smad pathway and to less extend through the p38 pathway, leading to the increase of the tendon markers and matrix proteins such as Scleraxis, Collagen I, and aggrecan but not osteogenic and only slightly Sox9 as a chondrogenic marker. Considering these points at the cellular perspective, BMP-7 seems to be a promising growth factor for the augmentation of tendon healing in clinics. It is interesting to note that BMP-7 induces a selective increase of BMP signalling components: BMPR-II, BMPR-Ib, and Smad8. The detailed analysis of the canonical and non-canonical signalling pathways suggests that BMP-7 supports the tenogenic phenotype, which is mediated primarily through Smad8 and to less extend through p38 associated pathways, without induction of osteogenic or chondrogenic phenotype.

## Material and Methods

### Cell culture

HTLCs of torn supraspinatus tendon biopsies were isolated from 6 male donors (62–67 years, Goutallier grade 1 or 2), undergoing arthroscopic or open shoulder surgery. Biopsies were obtained 3 to 5 mm from the torn proximal tendon edge. All patients gave their written informed consent. The tissue harvest was carried out in accordance with the local guidelines of the Charité-Universitätsmedizin Berlin and the study was approved by the ethics committee of the Charité-Universitätsmedizin Berlin (Ethic number: EA1/060/09).

HTLCs were isolated from SSP tendon biopsies by collagenase digestion (0.3% collagenase type CLS II) as described previously^56^. Cells were cultured with normal growth medium (Dulbecco’s Modified Eagle Medium (DMEM)/Ham’s F12 with 10% fetal calf serum (FCS) and 1% Penicillin/Streptomycin (P/S), all Biochrom AG. Germany) at 37 °C, with 95% humidity and 5% carbon dioxide, with a medium change three times per week.

### BMP-7 stimulation

Human tenocyte like cells (hTLCs) at passage 1 were thawed and cultured for 1 week prior the trypsinisation and then seeded in 35 mm petri dishes at a density of 4.5 × 10^4^ vital cells. The cells were cultivated for 3 days in the dishes until BMP-7 stimulation (rhBMP-7, R&D Systems GmbH, Germany). Medium was removed and medium without FCS was added and incubated for 3 h at 37 °C for starvation. Starving medium was removed and fresh starving medium supplemented with 200 or 1000 ng/ml rhBMP-7 was applied to the cells. The concentration is based on previous experiments^6^. After 15, 30, 60 and 120 min cells were lysed in 200 μl 1x Laemmli buffer (62.5 mM Tris HCl. 7.5% glycerol, 2.1% β-mercaptoethanol, 4.2% SDS. 0.01% bromphenolblue) and stored at −20 °C until used for Western Blot analysis. After 1, 2, 4 and 8 h RNA was isolated from the cells using the NucleoSpin RNA Kit (Macherey Nagel, Germany) for gene expression analysis by quantitative real-time polymerase chain reaction (qRT-PCR). Each experiment was made with n = 6 tenocyte cultures per group and time point.

To characterise changes in the cell phenotype after BMP-7 stimulation qRT-PCR was performed at the end of the stimulation experiment (time point: 8 h) for markers of tenocytes (scleraxis, mohawk, and collagen I), osteocytes (osteocalcin, osterix, and Runx2) and chondrocytes (Sox9, collagen II, and aggrecan) and compared to the unstimulated control.

Inhibition experiments were performed to clarify which intracellular pathways are actively induced by BMP-7 in tenocytes. Three different inhibitors were used having different effects on the Smad- and non-Smad pathways. LDN193189 (Cayman, USA), which was added in a concentration of 1 μM and 5 μM, inhibits SMAD1/5/8 phosphorylation by the BMP- type I receptors. Akt-inhibitor (Calbiochem, USA) is a selective inhibitor of the protein kinase B (AKT) (used concentration: 5 μM and 10 μM) and PD169316 (Cayman, USA) selectively inhibits p38 MAPK (used concentration: 5 μM and 10 μM). Tenocytes were starved for 3 h, the inhibitors added during the last starving hour, followed by a stimulation with BMP-7 (200 ng) and the inhibitors in different concentrations for further 8 hours. At the end of the experiment RNA was extracted and qRT-PCR performed. The experiments were performed in triplicates.

### qRT-PCR

RNA quantity and purity was analysed with Nanodrop ND-1000 Spectrophotometer (PeqLab Biotechnologie, Germany). A total of 100 ng RNA were transcribed into complementary DNA (cDNA) using the qScript cDNA Supermix (Quanta BioSciences. USA) with the Epgradient Mastercycler (Eppendorf. Germany). PCR was performed using 1.25 ng cDNA and the Sybr Green Mastermix (Quanta BioSciences). All primer sequences were designed using Primer 3 software (Freeware; http://frodo.wi.mit.edu/primer3) and were produced by Tib Molbiol, Germany (Primer sequences see Table 2). The CT value of the gene of interest was normalised to the CT of the housekeeping gene 18S ribosomal RNA and the primer efficiency was included in the calculation to optimise the validity of results^57^. The mean normalised expression (MNE) was calculated according to the following formula (Equation 1):


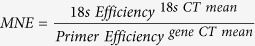


Gene expression rates were then calculated by using the negative control as a reference according to the formula (Equation 2):


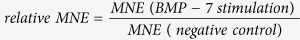


18S rRNA was selected based on the lowest regulation in response to BMP-7 stimulation when compared to other reference genes that were tested, i.e. Glyceraldehyde 3-phosphate dehydrogenase (GAPDH), Hypoxanthine phosphoribosyl transferase (HPRT), and 60S ribosomal protein L13 (RPL13).

### Western Blotting

Cells were lysed with 1x Laemmli-buffer and stored at −20 °C until used for Western Blot analysis. Proteins were detected by SDS-PAGE (Mini-PROTEAN Tetra Cell, BioRad) and western blotting (Trans-Blot^®^ Cell, BioRad) using antibodies for the Smad-pathway: total Smad1 and the combined antibody for p-Smad1/5/8, as well as p-p44/42 MAPK (Erk1/2), p-SAPK/JNK, p-Akt and p-p38 for the non-Smad pathways in combination with species-specific IRDye^®^-conjugated secondary antibodies (Table 3). GAPDH and β-Actin served as loading controls. Imaging was conducted using the Odyssey imager and LiCor Odyssey software. (n = 6). For normalisation the bands of the p-Smad1/5/8 and total Smad1 were normalised to GAPDH (including background correction). In a second step the ratio of the normalised p-Smad1/5/8 and normalised total Smad1 was calculated. The non-Smad signalling proteins were only analysed exemplarily (n = 3 different donors). Bands from the protein of interest were normalised to β-Actin or GAPDH and the unstimulated control.

### Statistics

The results are given in box plots as median with 25 and 75 percentiles. Statistical analysis was done using SPSS 20 (IBM, USA). Depending on the sample size, significant differences were analysed with the Mann-Whitney U test for non-parametric data to compare the stimulation groups with the untreated control and adjusted with the Bonferroni-Holm correction (n = 6) or with a one-way ANOVA corrected by Dunnett´s test to control or BMP-7 treatment (n = 3). Differences were considered significant at the values of p < 0.05.

## Additional Information

**How to cite this article**: Klatte-Schulz, F. *et al*. An investigation of BMP-7 mediated alterations to BMP signalling components in human tenocyte-like cells. *Sci. Rep.* **6**, 29703; doi: 10.1038/srep29703 (2016).

## Figures and Tables

**Table 1:** Basal gene expression levels (CT-values) of unstimulated hTLCs.

	Tenogenic marker			Osteogenic marker			Chondrogenic marker		
	Mohawk	Scleraxis	Collagen I	Osteo calcin	Runx2	Osterix	Aggrecan	Sox9	Collagen II
Median	26.81	27.39	19.12	31.41	n.d.	n.d.	23.76	34.14	n.d.
1.-3. quartile	26.32–27.60	27.16–28.59	18.29–19.32	30.84–31.93			23.35–25.74	33.50–34.94	
	**Type I receptors**			**Type II receptors**			**R-Smads**		
	**BMPR-Ia**	**BMPR-Ib**	**ActR-I**	**BMPR-II**	**ActR-IIa**	**ActR-IIb**	**Smad1**	**Smad5**	**Smad8**
Median	26.49	31.52	27.41	25.23	34.53	31.69	28.84	26.58	28.45
1.-3. quartile	26.22–26.95	30.79–32.16	27.12–27.80	25.04–25.67	33.62–36.08	31.01–32.18	28.56–29.17	26.29–26.92	28.06–28.88

**Table 2:** Primer Sequences.

Gene	Accession number	Primer sequence Forward	Primer sequence Reverse	Efficiency
18S	NM_022551	CGGAAAATAGCCTTTGCCATC	AGTTCTCCCGCCCTCTTGGT	1.79
BMPR-Ia	NM_004329.2	TGCTGGACGAAAGCCTGAAC	TTCCACGATCCCTCCTGTGA	1.78
BMPR-Ib	NM_001203.2	ATGCCACCACCATTGTCCAG	GTGACCACAGGCAACCCAGA	2.05
ActR-I	NM_001111067.2	GTCGGGAAAGGCAGGTATGG	TCCGTTTCCCTGAACCATGA	1.93
BMPR-II	NM_001204	GCTCTTGCCGTCTTGCTCAT	GGCGCACCAGTCTATTTCCA	1.93
ActR-IIA	NM_001278580	TTGCGGGGATTGTCATTTGT	GGAGAAGGTGGGGGTGGTC	2.03
ActR-IIB	NM_001106	CTGCACTGCTACGCCTCCTG	ACACCTGGGGGTTCTCCTCA	1.98
Smad1	NM_005900.2	GAAGCGTTCCATGCCTCCTC	GGCATACACCTCCCCTCCAA	1.75
Smad4	NM_005359	TCCCAACATTCCTGTGGCTTC	CTGCTGCTGTCCTGGCTGA	2.00
Smad5	NM_001001420.1	TTCCACCAGCCCAACAACAC	GGCAGGAGGAGGCGTATCAG	1.91
Smad8/9	NM_001127217	ACACCACCCCTGCCTTATCA	CCTGGAATGTCTCCCCAACTC	1.81
ID1	NM_181353	GCTGCTCTACGACATGAACG	CCAACTGAAGGTCCCTGATG	1.87
OC	NM_199173.4	TGAGAGCCCTCACACTCCTC	CGCCTGGGTCTCTTC ACT AC	1.78
Col-IA1	NM_000088.3	TGACCTCAAGATGTGCCACT	ACCAGACATGCCTCTTGTCC	1.92
Col-IIA1	NM_033150.2	CGCACCTGCAGAGACCTGAA	TCTTCTTGGGAACGTTTGCTGG	1.87
Runx2	NR_103532	GCCCCCAAACAGTATCTTGA	GCCTGAAGTGAGGTTTTAGGC	1.86
Aggrecan	NM_001135.3	CCAGTGCACAGAGGGGTTTG	TCCGAGGGTGCCGTGAG	1.89
Mohawk	NM_173576.2	TGGTTTGCTAATGCAAGACG	CCTTCGTTCATGTGGGTTCT	1.85
Osterix	NM_001173467.2	CACCCACCTCAGGCTATGCT	TGGATGCCTGCCTTGTACCA	2.08
Sox9	NM_000346	GACTCGCCACACTCCTCCTC	CTCAGCTCGCCGATGTCCA	1.84
Scleraxis	Commercial product. QuantiTect Primer Assay (Qiagen)			

**Table 3:** Antibodies for western blot.

Name	Species	Manufacturer	Dilution
GAPDH (14C10) (#2118)	Rabbit mAb	Cell signaling	1:2000
beta Actin (Clone AC-15)	Mouse mAB	Sigma Aldrich	1:5000
total Smad1 (#6944)	Rabbit mAb	Cell signaling	1:1000
p-Smad1/5/8 (#9511)	Rabbit mAb	Cell signaling	1:1000
p-p44/42 MAPK (Erk1/2) (#9101)	Rabbit pAb	Cell signaling	1:1000
p-Akt (Ser473) (D9E) XP^®^	Rabbit mAb	Cell signaling	1:1000
p-SAPK/JNK (Thr183/Tyr185) (81E11)	Rabbit mAb	Cell signaling	1:1000
Anti-ACTIVE^®^ p38 (pTGpY)	Rabbit pAb	Promega	1:1000
IRDye 800CW polyclonal Anti-Rabbit IgG	Goat pAb	Li-Cor Biosciences	1:15000
IRDye^®^ 680RD anti-Mouse IgG	Goat pAb	Li-Cor Biosciences	1:15000
